# Location, location, location: subcellular protein partitioning in proteostasis and aging

**DOI:** 10.1007/s12551-021-00890-x

**Published:** 2021-11-19

**Authors:** Anita V. Kumar, Louis R. Lapierre

**Affiliations:** Department of Molecular Biology, Cell Biology and Biochemistry, Brown University, Providence, RI 02912 USA

**Keywords:** *C. elegans*, Nucleocytoplasmic partitioning, Proteostasis

## Abstract

Somatic maintenance and cell survival rely on proper protein homeostasis to ensure reliable functions across the cell and to prevent proteome collapse. Maintaining protein folding and solubility is central to proteostasis and is coordinated by protein synthesis, chaperoning, and degradation capacities. An emerging aspect that influences proteostasis is the dynamic protein partitioning across different subcellular structures and compartments. Here, we review recent literature related to nucleocytoplasmic partitioning of proteins, nuclear and cytoplasmic quality control mechanisms, and their impact on the development of age-related diseases. We also highlight new points of entry to modulate spatially-regulated proteostatic mechanisms to delay aging.

## Introduction

One of the key hallmarks of aging is the loss of protein homeostasis (Lopez-Otin et al. 2013; Moreno and Aldea 2020), which has global impact on cellular function, flexibility, resilience, and survival. Our current understanding of conserved molecular mechanisms of proteostasis and aging has greatly benefited from studies in model organisms including yeast (*Saccharomyces cerevisiae*), nematodes (*Caenorhabditis elegans*), and flies (*Drosophila melanogaster*) (He et al. 2018; Kenyon 2010; Yu and Hyun 2021). Several key lifespan-modulating pathways have been identified, including nutrient and germline signaling, mitochondrial respiration, and translation attenuation (Denzel et al. 2019). Different genetic and environmental longevity interventions have displayed proteostatic enhancements, but our understanding of the impact of these cellular improvements on proteome dynamics during aging remains incomplete. One of the major progressive changes associated with aging is the loss in solubility for numerous proteins, which jeopardizes the stability of the whole proteome (David et al. 2010; Reis-Rodrigues et al. 2012; Walther et al. 2015) and may form the basis of many age-related diseases. Protein chaperones can delay the collapse of the proteome by mitigating the impact of protein aggregation (Ben-Zvi et al. 2009) and by attempting to refold misfolded proteins. Cells have two major protein degradation processes, the 26S proteasome and the autophagy/lysosome pathway, that can preventively degrade misfolded, damaged, and aggregating proteins (Dikic 2017). Long-lived nematodes show enhanced proteasome function (Vilchez et al. 2012) and increased autophagic flux (Lapierre et al. 2015). Notably, 26S proteasomes may degrade up to 90% of intracellular proteins (Lee and Goldberg 1998). Organellar proteases can also contribute to proteostatic quality control (Quiros et al. 2015; Sun and Brodsky 2019). Altogether, the efficiency of these degradation pathways governs the ability of cells to prevent the accumulation of damaged and aggregating proteins, thereby maintaining protein solubility and function necessary for cell survival.

During the process of aging, protein solubility progressively wanes (Hipp et al. 2019; Vecchi et al. 2020) and several proteins aggregate (David et al. 2010; Reis-Rodrigues et al. 2012; Walther et al. 2015) as protein degradation efficiency fades and chaperoning systems are overwhelmed. Age-associated protein aggregation arises even in longevity models (Walther et al. 2015), but the types and properties of proteins aggregating as well as the quantity of aggregate-associated chaperones differ between wild-type and long-lived animals (Walther et al. 2015). This intriguing observation is in line with other studies showing that aggregation can serve a protective role (Cohen et al. 2009; Saad et al. 2017) and is part of the arsenal of tools cells employ to minimize cellular dysfunction associated with unstable proteomes. Subcellular protein repartitioning appears to underlie the ability of cells to withstand the proteome destabilization associated with heat stress (Domnauer et al. 2021). Subcellular localization of unstable proteins in the cell dictates their propensity to aggregate. Indeed, cytoplasmically accumulating proteins have a higher likelihood to aggregate than those accumulating in the nucleus (Samant et al. 2018), suggesting that supersaturation barriers and aggregation dynamics, as well as chaperoning capacity, are compartment-specific. Another example is the expression of unstable proteins in the ER reduces their propensity to aggregate (Vincenz-Donnelly et al. 2018). This location-specific nature of protein aggregation and toxicity provides a mechanism by which cells can regulate overall proteome stability by modulating subcellular protein partitioning.

**Fig. 1:**
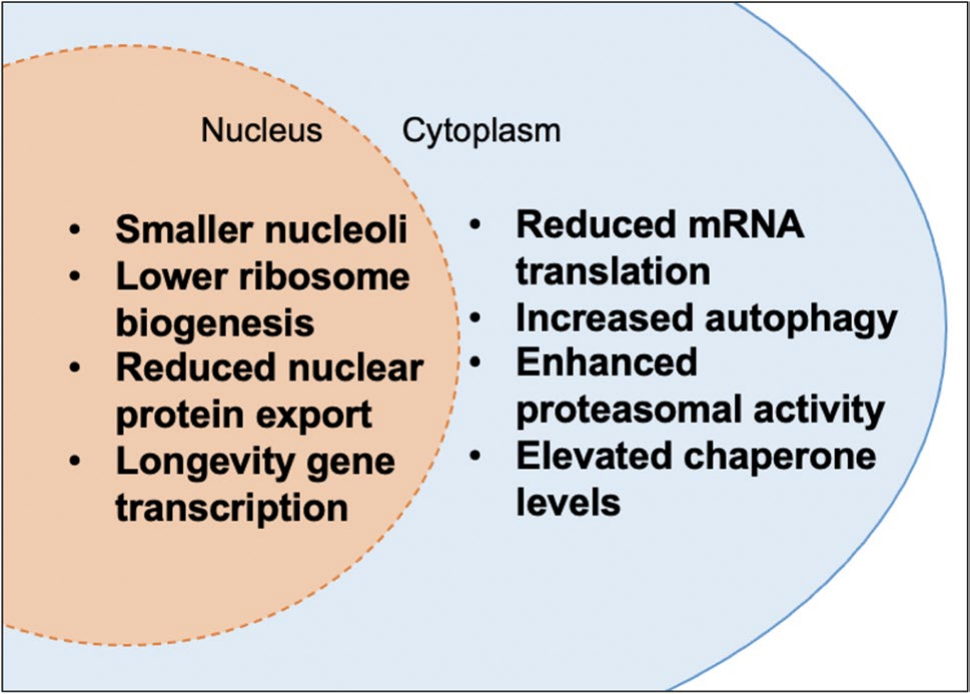
Key proteostatic mechanisms associated with longevity

Long-lived nematodes display a variety of nucleocytoplasmic proteostatic improvements that impact proteome stability and enable lifespan extension (Fig. 1). These include modulation of ribosomal function (Hansen et al. 2007; Schosserer et al. 2015; Tiku et al. 2017) as well as reduced protein export into the cytoplasm (Silvestrini et al. 2018). Transcriptional activation of proteasome (Li et al. 2011; Vilchez et al. 2012) and autophagy genes (Lapierre et al. 2013) as well as chaperones (Murphy et al. 2003) via longevity-associated transcription factors (including, but not limited to DAF-16/FOXO, HLH-30/TFEB, SKN-1/NRF2, HSF-1/HSF1) (Denzel et al. 2019) improve cytoplasmic proteostasis (Fig. 1). Altogether, these proteostatic changes prevent protein supersaturation and decrease the burden on chaperones and protein degradation machineries, which in turn delay the progressive solubility decline associated with neurodegenerative diseases and aging (Ben-Gedalya and Cohen 2012; Ciryam et al. 2013).

From synthesis to degradation, proteins are constantly surveilled for proper folding and damage, but their dynamic subcellular partitioning, preponderance in different compartments, and association with various organelles leads to a variety of proteostatic outcomes that have important ramifications on disease onset and progression, and ultimately on aging itself. Therefore, spatio-temporal regulation of proteostasis is key in somatic maintenance and health (Sontag et al. 2017). This review highlights subcellular mechanisms of proteostasis and their impact on longevity and aging, with an emphasis on protein trafficking across the nuclear pore as well as specific nuclear and cytoplasmic proteostatic mechanisms.

## Nucleocytoplasmic protein trafficking

Proteome partitioning between the cytoplasm and the nucleus is mediated by passive and facilitated transport of proteins across the nuclear pore (Knockenhauer and Schwartz 2016; Timney et al. 2016). The nuclear pore is a massive complex (120 MDa in humans) in the nuclear membrane consisting of about 30 different nuclear pore proteins, or nucleoporins, in numerous copies (D’Angelo and Hetzer 2008). Altogether, the nuclear pore structure includes a ring-like pore, a nuclear basket, and cytoplasmic filaments (Solmaz et al. 2013), and integrates between 500 and 1000 nucleoporin proteins (Beck and Hurt 2017; Knockenhauer and Schwartz 2016; Schwartz 2016). Some of the nuclear pore proteins have particularly long lifespan in the nuclear pore and are exchanged at a low rate (Toyama et al. 2013), suggesting that damage in these proteins may result in lasting destabilization of the nuclear pore. Indeed, with age, nuclear pore complex instability and permeability progressively increases, leading to mislocalization of several proteins, a phenomenon that is prevented in long-lived nematodes (Doucet et al. 2010). Altogether, these studies suggest that maintenance of the nuclear pore integrity is essential for longevity (Toyama and Hetzer 2013).

**Fig. 2:**
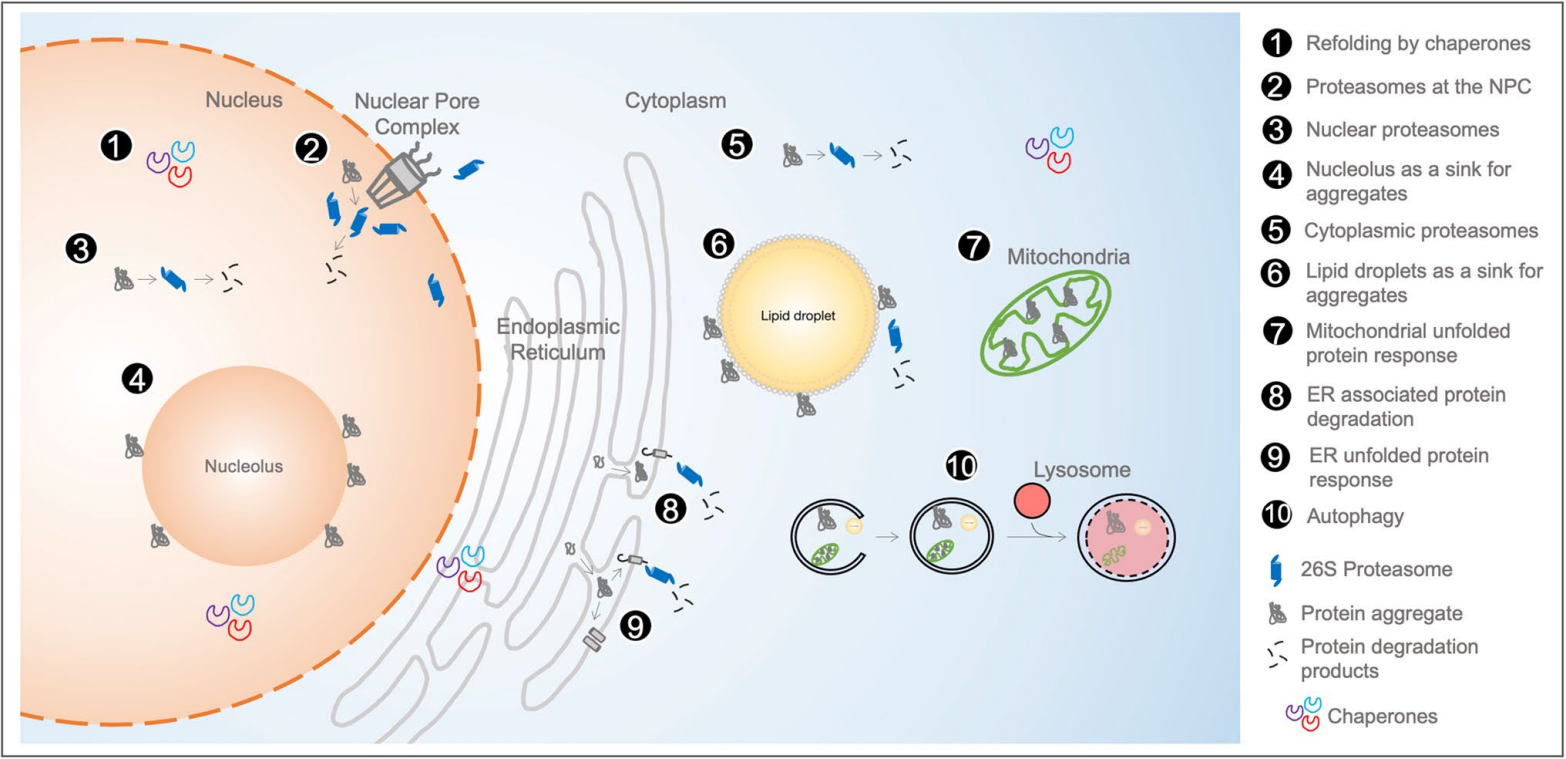
Subcellular locations relevant for proteostasis

The accepted passive threshold across the nuclear pore is 40 kDa (Knockenhauer and Schwartz 2016; Schmidt and Gorlich 2016) and transporters called karyopherins can recognize and facilitate the traffic of larger proteins across the nuclear pore, a process involving Ran GTPases (Cavazza and Vernos 2015). The partitioning of large proteins (> 40 kDa) between the nucleus and the cytoplasm involves a specific recognition of sequences within cargo proteins by karyopherins. The karyopherin family of proteins consists of trafficking receptors named importins (18 in humans) and exportins (6 in humans). Importins recognize nuclear localization sequences of cytoplasmic proteins and mediate their transit from cytoplasm to nucleus. Exportins recognize nuclear export sequences of nuclear-localized proteins and facilitate their transport from the nucleus to the cytoplasm. To maintain transport capacities across the nuclear pore, karyopherins are returned to their relevant site of action after trafficking. This dynamic cycle of import and export governs the temporal specification of nuclear and cytoplasmic proteomes, and ultimately impacts an array of key cellular processes including pathways associated with aging (Fig. 2).

**Fig. 3:**
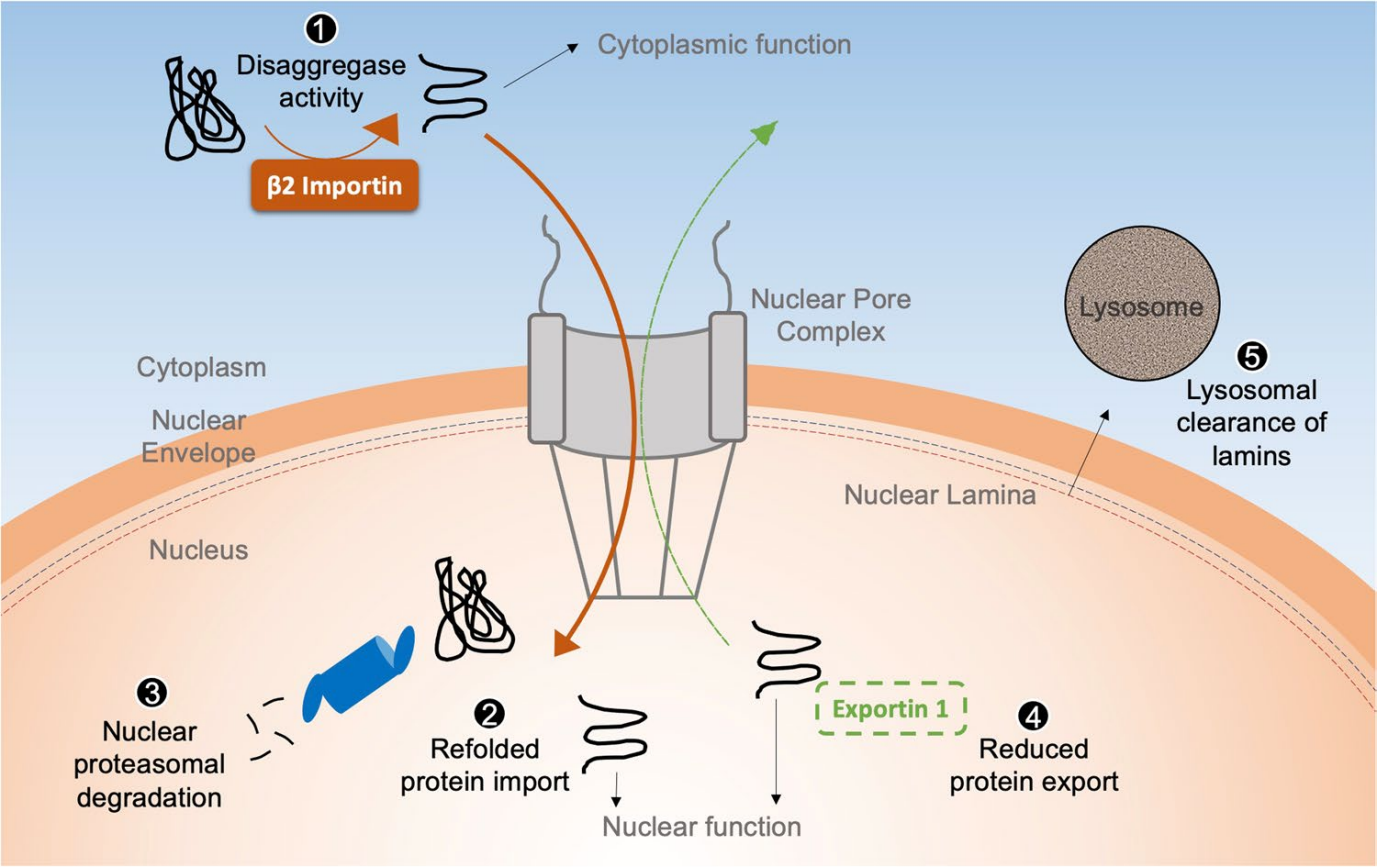
Coordination of nucleocytoplasmic protein partitioning and proteostatic mechanisms

Several diseases have a fundamental basis in nucleocytoplasmic transport dysfunction, including cancer (Gandhi et al. 2018), neurodegeneration (Zhang et al. 2015), and age-related diseases (Kim and Taylor 2017). Many neurodegenerative diseases are characterized by impairments in nucleocytoplasmic protein partitioning (Kim and Taylor 2017) and nucleolar dynamics (White et al. 2019), in addition to autophagic defects due to lysosomal dysfunction (Wong and Cuervo 2010). Since intracellular mislocalization of proteins can lead to deleterious compartmental loss-of-function(s) or predispose mislocalized cargo proteins to aggregate and impair proteostatic mechanisms, nuclear transport dysfunction may be a factor underling the onset of neurodegeneration (Kim and Taylor 2017). Defective nucleocytoplasmic partitioning has been linked to the development of ALS as RNA processing protein, TDP43, aberrantly distributes in the cytoplasm (Solomon et al. 2018). In nuclei of cells from Hutchinson-Gilford progeria syndrome patients, mutated lamins that normally would provide structural support aberrantly accumulate, resulting in genomic instability, a feature exacerbated by dysfunctional nuclear protein transport (Kelley et al. 2011). Phosphorylated form of the protein Tau (Bejanin et al. 2017), a pathologically relevant agent in AD, was recently found to interact with nuclear pore components and disrupt nuclear protein transport leading to cytosolic protein mislocalization, which in turn facilitated cytosolic Tau aggregate formation (Eftekharzadeh et al. 2018). Studies of different proteinopathies have identified key importins and exportins as modifiers of the onset of these diseases (Fig. 3).

Karyopherin β2, an importin involved in the import of several nuclear localization sequence-containing RNA-binding proteins (Chook and Suel 2011), was recently shown to display chaperoning and disaggregase functions for unstable proteins (FUS, dipeptide repeats, etc.) that agglomerate in the cytoplasm and are relevant in ALS and FTD (Guo et al. 2018; Hutten et al. 2020; Robinson et al. 2020). This pre-import disaggregation function demonstrates that repartitioning of certain RNA-binding proteins from the cytosol to the nucleus may improve the solubility and function of proteins that are bound to aggregate and mediate proteotoxicity in the cytoplasm. In turn, this chaperone-like function likely reduces the proteostatic burden in the cytoplasm, in addition to refolding and compartmentalization of unstable proteins in order to restore their native structure and function. Alternatively, unstable protein import in the nucleus may facilitate their degradation via nuclear 26S proteasomes (Albert et al. 2017).

Exportin 1 (XPO1/CRM1), an exportin involved in the recognition and transport of potentially hundreds of nuclear export sequence-containing proteins (Kirli et al. 2015), has been linked to the control of the longevity-associated pathway of autophagy in *C. elegans* (Kumar et al. 2018; Silvestrini et al. 2018). XPO1 is a highly conserved nuclear export receptor involved in trafficking several proteins including translation factors, vesicle coat proteins, centrosomal and autophagy proteins, ubiquitin pathway proteins, and ribosomal subunits out of the nucleus (Kirli et al. 2015). Notably, XPO1 levels are elevated in various cancers leading to nuclear depletion of tumor-suppressing proteins (Gandhi et al. 2018). XPO1 is also involved in snoRNA (Boulon et al. 2004) and snRNP trafficking (Sleeman 2007) and can localize to the nucleolus where it plays a role in rRNA processing (Bai et al. 2013). Recently developed XPO1 inhibitors showed success in slowing tumorigenesis in a variety of cancers and the selective inhibitor of nuclear export (SINE), Selinexor (KPT-330, Karyopharm Therapeutics), was approved for relapsed multiple myelomas in 2020 (Chen et al. 2018). Inhibition of XPO1 using SINE leads to nucleocytoplasmic repartitioning of several proteins and a corresponding reduction in translation rate (Wahba et al. 2018). This is associated with improvement in the process of autophagy and lysosomal biogenesis via the nuclear enrichment of the autophagy transcriptional regulator TFEB (Silvestrini et al. 2018), improving proteostasis and increasing lifespan in nematodes and ALS-afflicted flies (Silvestrini et al. 2018; Zhang et al. 2015). XPO1 inhibition was also recently shown to mitigate the nuclear defects of progeria (Garcia-Aguirre et al. 2019), suggesting that reducing protein export can foster healthy nuclear structure and proteome.

Overall, the dynamics of nucleocytoplasmic protein partitioning is an emerging field with promising potential to markedly enhance our understanding of the process of aging and the onset of age-related diseases. Moreover, the development of new selective inhibitors of karyopherins is bound to improve our ability to pharmacologically modify the partitioning of proteins in cells in order to modulate proteostasis by leveraging different proteostatic mechanisms in the nucleus and the cytoplasm.

## Nuclear proteostasis: nexus of ribosomal subunit and protein quality control

The nuclear proteome is diverse and requires proper protein surveillance in order to maintain nuclear structure and dynamic processes that characterize this essential organelle (Enam et al. 2018; Shibata and Morimoto 2014). As cellular proteome is specified by ribosomes, proper assembly of pre-ribosome subunits in the nucleus ultimately governs the rate of mRNA translation. Ribosome assembly originates inside the nucleus in the membraneless nucleolus (Boisvert et al., 2007; Iarovaia et al. 2019), where different ribosomal RNAs (rRNA) are transcribed by RNA polymerases (Paule and White 2000) and processed into the 40S (18S rRNA + 33 ribosomal proteins) and 60S (5S, 5.8S, and 28S rRNA + 46 ribosomal proteins) ribosomal subunits (Pena et al. 2017). Processing of pre-rRNA is required for proper ribosomal subunit assembly and is promoted by the highly conserved rRNA 2’O-methyltransferase fibrillarin (FIB-1/FBL) (Pereira-Santana et al. 2020). The nucleolus can expand or retract to address cellular needs for ribosomal biogenesis, and fibrillarin levels have been correlated with nucleolar expansion (Weber and Brangwynne 2015), which stimulates the rate of ribosome assembly (Tollervey et al. 1993). Interestingly, proteins that become unstable in the nucleus can accumulate inside nucleoli (Frottin et al. 2019). Ribosomal subunits that are translated in the cytoplasm require nuclear import to assemble with processed rRNAs. Subsequently, newly assembled rRNA-containing ribosomal subunits are exported out of the nucleus and combine to form large 80S ribosomes for mRNA translation. Notably, when exported in the cytoplasm, supernumerous ribosomal subunits (An and Harper 2020; Sung et al. 2016a, b), mislocalized (Yanagitani et al. 2017) and stalled ribosomes (Matsuo et al. 2017) can be sent for proteasomal and lysosomal degradation. Several long-lived nematodes display smaller nucleoli, in part via lower FIB-1, rRNA, and ribosomal protein levels, and specifically silencing *fib-1* extends lifespan in *C. elegans* (Tiku et al. 2017). High levels of FBL expression are found in several cancers (Koh et al. 2011; Marcel et al. 2013; Su et al. 2014) and nucleolar hypertrophy is a hallmark of poor tumor prognosis (Derenzini et al. 2009). An E3 ubiquitin ligase, NCL-1/TRIM2, negatively regulates FIB-1 levels (Tiku and Antebi 2018; Tiku et al. 2017; Yi et al. 2015). *TRIM2* mutations in humans are linked to axonal neurodegeneration (Ylikallio et al. 2013), and mutating *ncl-1* in long-lived nematodes restores their nucleoli to wild-type size and significantly impairs their longevity (Tiku et al. 2017).

Different environmental stresses, including nucleotide depletion, heat shock, hypoxia, or UV, generate a nucleolar stress response (Rubbi and Milner 2003; Yang et al. 2018). This response elicits a signaling cascade mediated in part by p53 (Nicolas et al. 2016), which results in nucleolar fragmentation and disruption, and is associated with issues in ribosome biogenesis. Another environmental stress, starvation, results in chaperones (heat shock proteins) repartitioning into the nucleus (Chughtai et al. 2001; Nollen et al. 2001). Aggregated nucleoplasmic proteins can accumulate in the nucleolus (Latonen 2019), in particular when proteasome function is compromised (Latonen et al. 2011). In yeast, acute heat stress leads to the reversible formation of nucleolar protein aggregates (Gallardo et al. 2020). The nucleolus is also a temporary store for epigenetic regulators during heat shock, which are subsequently functionally restored after recovery from heat stress (Azkanaz et al. 2019). Aggregates in the nucleus have also been found in depots called intranuclear quality control compartment (INQ) (Miller et al. 2015). Notably, nuclear aggregate accumulation has been linked to polyglutamine-induced disease such as Huntington’s disease (Klement et al. 1998; Schilling et al. 2004). Mutated ⍺-synuclein was also shown to trigger nucleolar stress in a murine model of Parkinson’s disease (Evsyukov et al. 2017). Interestingly, there are mechanistic links between nucleolar stress and autophagy (Pfister 2019), and nucleolar proteins can be degraded via nucleophagy (Mostofa et al. 2018).

Heat shock proteins serve as chaperones and are found in both the nucleus and the cytoplasm (Echtenkamp and Freeman 2014; Vabulas et al. 2010). They modulate protein aggregation by converting unstable proteins into their native fold or into manageable proteasome targets (den Brave et al. 2020). Cryo-electron microscopy imaging of the nuclear pore in the green alga *Chlamydomonas reinhardtii* demonstrated tethering and enrichment of 26S proteasomes at the nuclear basket side (Albert et al. 2017), suggesting that a quality control checkpoint for proteins exists for nuclear proteins that are trafficked across the nuclear pore (Fig. 3). Studies in the yeast *S. cerevisiae* showed that quality control of cytoplasmic and nuclear proteins is mediated by spatially specific E3 ubiquitin ligases (Gardner et al. 2005) with different preferences for ubiquitin linkages (Samant et al. 2018). Recently, a study demonstrated that the accumulation of selective autophagy receptor SQSTM1 in nuclear condensates, brought about by reducing nuclear protein export, improves proteasomal function and degradation of c-myc, a key regulator of ribosome biogenesis and nucleolar dynamics (Fu et al. 2021). Thus, nuclear localization of autophagy-related factors can modulate different proteostatic mechanisms and impact proteostasis globally. Altogether, these studies highlight the ability of cells to sequester nuclear proteins into condensates or around the nuclear pore in order to determine their fate.

## Cytoplasmic proteostasis: organelle-specific and bulk protein quality control

The cytoplasm encompasses several membrane-bound organelles that interact with each other and mediate and integrate key cellular functions (Cohen et al. 2018). As organisms age, organelles accumulate damage and need to be degraded. Bulk degradation of these organelles is mediated by the recycling process of autophagy and lysosomal degradation (Galluzzi et al. 2017; Lapierre et al. 2015). Selective sequestration of organelles is mediated by selective autophagy receptors that recognize damaged organelles and facilitate their degradation (Zaffagnini and Martens 2016). For instance, efficient degradation of mitochondria via mitophagy is required in the lifespan extension of long-lived nematodes (Palikaras et al. 2015). Concomitantly, cytoplasmic 26S proteasomes degrade a vast array of damaged and ubiquitinated proteins. When proteostatic and protein degradation machineries are overwhelmed, aggregating proteins can accumulate in specific sites in the cytoplasm called the insoluble protein depot (IPOD) and the juxtanuclear quality control (JUNQ) compartments (Samant et al. 2018), akin to the originally described aggresomes (Johnston et al. 1998). In specific proteostatic challenges, cells can activate organelle-specific unfolded protein responses (UPR^ER^ or UPR^MT^) (Shpilka and Haynes 2018; Walter and Ron 2011), which results in enhancement in protein folding in order to ensure solubility and function. Aging leads to dysfunction in UPR at the endoplasmic reticulum (UPR^ER^) (Frakes and Dillin, 2017) and mitochondrial UPR (UPR^MT)^, which can affect stem cells and tissue aging (Mohrin et al. 2015). The ER and mitochondria also possess lumenal proteases that directly degrade proteins (synthesized or imported) (Quiros et al. 2015; Sun and Brodsky 2019). In yeast, mitochondria can degrade resident proteins (Hughes et al. 2016) and aggregating proteins imported from the cytoplasm (Zhou et al. 2014). The ER can also send proteins to the proteasome via ER-associated degradation (ERAD) where polypeptides are recognized and threaded back into the cytosol via a retro-translocon (Brodsky 2012; Qi et al. 2017). Lipid droplets can serve as an intermediary organelle for ERAD where cargo bound for degradation transit on the lipid droplet surface before being degraded by the proteasome (Olzmann and Carvalho 2019).

The endosomal sorting complexes required for transport (ESCRT) is a multisubunit complex tasked with sorting ubiquitinated proteins and multi-vesicular bodies toward lysosomal degradation (Schmidt and Teis 2012). Compromised ESCRT leads to the autophagic dysfunction and accumulation of aggregating proteins relevant to neurodegeneration (Oshima et al. 2016). Notably, proteins associated with the lysosomal membrane can be degraded by lysosomes via the ESCRT machinery (Zhu et al. 2017) or intraluminal fragments (McNally and Brett 2018). Overall, the cytoplasm possesses several options to stabilize or degrade proteins, but aging systematically decreases the ability of this compartment to properly manage proteostasis, resulting in molecular crowding and aggregated protein deposition.

## Conclusion

**Fig. 4:**
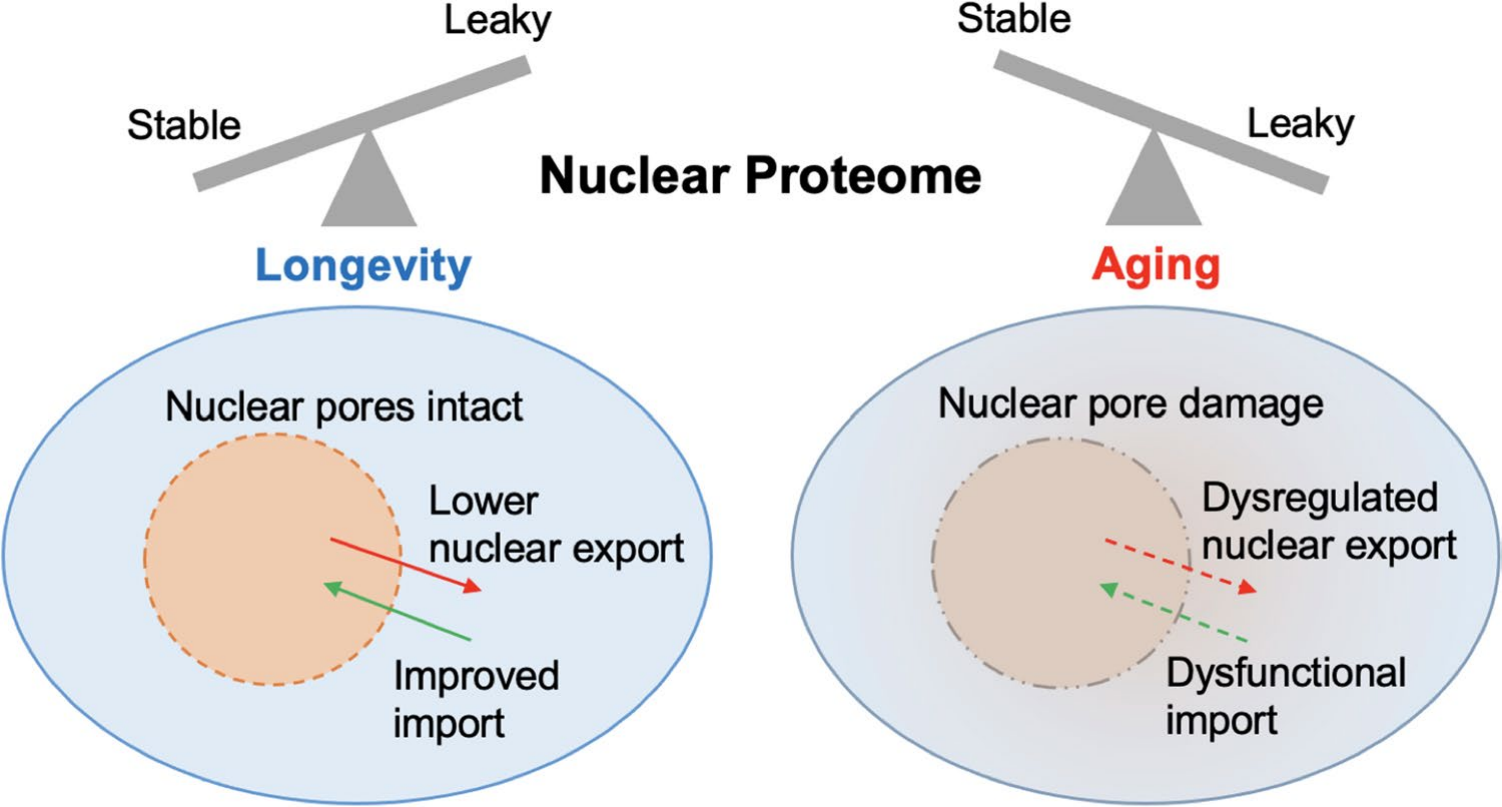
Nucleocytoplasmic proteostatic inter-relationship during aging

Cells employ an arsenal of mechanisms to maintain protein homeostasis in order to ensure cell survival and to adapt to changing environments. In addition to compartment-specific proteostatic processes, the integration of different mechanisms (such as nucleolar dynamics and autophagy (Pfister 2019)) generates a global response against proteotoxic stress associated with aging. Signaling between different organelles, such as mitochondria and nucleus (Fang et al. 2016), and also between tissues may serve to generate organismal response to stress and aging (Zhang et al. 2018). Signaling pathways that can coordinate a proteostatic response, such as nutrient signaling mediated by mTOR complexes (Laplante and Sabatini 2012) and the integrated stress response via the eIF2 complex (Costa-Mattioli and Walter 2020), are important mechanisms to balance protein synthesis and degradation. These processes modify ribosome biogenesis and function, protein specification, and localization, and ultimately affect the stability of the proteome. An important mechanism of proteostasis that potentially fails during aging is the proper partitioning of proteins across the nuclear pore (Fig. 4). Mislocalization of proteins fosters aggregation, but concomitant aberrant DNA release into the cytoplasm can also lead to inflammation and neurodegeneration (Paul et al. 2021). Therefore, pharmacologically modulating the nucleocytoplasmic partitioning of proteins is emerging as an attractive strategy to impact the stability of the whole proteome and delay aging.
